# Rapid Diagnosis of Infective Endocarditis in the Emergency Department: A Case Series Highlighting the Role of Point-of-Care Ultrasound

**DOI:** 10.7759/cureus.87236

**Published:** 2025-07-03

**Authors:** Jonathan Schonert, Joseph Minardi, Zouyan Lu

**Affiliations:** 1 Emergency Medicine, St. Luke's Hospital, Chesterfield, USA; 2 Emergency Medicine, West Virginia University, Morgantown, USA; 3 Infectious Disease, Oregon Health & Science University, Portland, USA

**Keywords:** endocarditis, intravenous drug abusers (ivda), point-of-care ultrasound (pocus), ultrasound, vegetation

## Abstract

Infective endocarditis can be a challenging and potentially life-threatening diagnosis in the emergency department (ED) due to its often nonspecific presentation. Missed or delayed recognition can result in serious complications. Point-of-care ultrasound (POCUS) is increasingly utilized in emergency medicine to expedite the diagnosis of critical conditions, including endocarditis.

We present a case series of four patients who presented to the ED with varied chief complaints - altered mental status, fever with shortness of breath, delirium, and isolated extremity pain. In each case, emergency physicians identified valvular vegetations using POCUS, prompting expedited consultation and management. Each case involved a different cardiac valve, with representative images and video clips provided. We outline our bedside approach and highlight key sonographic findings to aid in the early identification of infective endocarditis.

Emergency physicians are increasingly equipped to identify life-threatening pathology with bedside imaging. These cases underscore how POCUS can aid in the early diagnosis of infective endocarditis, especially when presentations are atypical. Rapid identification can lead to earlier initiation of antibiotics, targeted diagnostic workups, and specialist involvement, leading to improved patient outcomes.

## Introduction

Infective endocarditis (IE) remains a challenging diagnosis in the emergency department (ED) due to its often nonspecific presentation [1]. Many patients exhibit symptoms such as fever, malaise, and headache, which are common presentations for a large majority of ED patients. Missed or delayed recognition can result in serious complications, including worsening valvular regurgitation, heart failure, embolic events, and sepsis [2]. The mortality rate for IE remains high, particularly in cases with delayed intervention [3].

Managing these patients often requires a multidisciplinary team, including cardiothoracic surgery, infectious disease, and sometimes addiction medicine [4] - resources that may not be readily available in all hospitals, especially in rural or community settings. Early identification of IE in the ED is crucial, as it allows for timely initiation of antibiotics, risk stratification, and appropriate transfer to tertiary care centers when needed [5]. Given current challenges with ED boarding and prolonged transfer delays, early recognition using point-of-care ultrasound (POCUS) may also help identify cases that should be prioritized for transfer to tertiary care settings.

POCUS is increasingly used in emergency medicine to expedite the diagnosis of critical conditions, including endocarditis [1]. In patients presenting with fever and no obvious source - an initial presentation for many IE cases [6] - POCUS can provide valuable diagnostic clues that guide early management decisions. This case series highlights four patients who presented to the ED with varying symptoms and were found to have IE using POCUS. In each case, early ultrasound findings facilitated a more rapid diagnosis, allowing for timely interventions and disposition decisions. These cases illustrate a spectrum of presentations, some more clinically apparent than others, but all reinforcing the value of POCUS as an early diagnostic tool to streamline workup and optimize treatment strategies for suspected IE. We also describe our approach to utilizing POCUS for the evaluation of endocarditis in the emergency setting.

## Case presentation

Case 1

A 30-year-old male presented to a rural ED with confusion. Due to his altered mental status, history was limited. His vital signs were heart rate (HR) 133, blood pressure (BP) 144/81, respiratory rate (RR) 18, temperature 37.0°C, and SpO₂ 99%. There were no focal neurologic deficits, but he appeared agitated. Physical examination revealed red lesions on his hands, splinter hemorrhages of the nails, and petechial rash on the trunk. No heart murmurs were appreciated. Notably, track marks were observed on his forearm, raising suspicion for IE.

**Figure 1:**
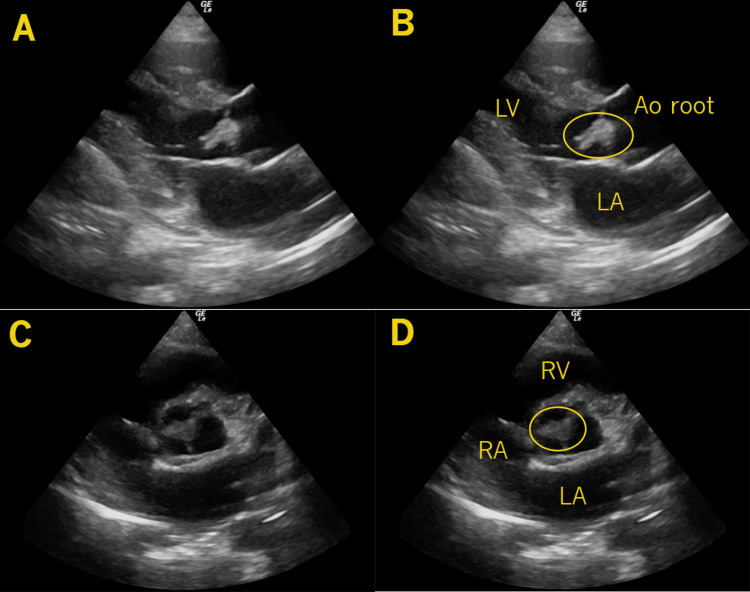
Aortic Valve Vegetation In these images, a large mass (circle) is seen attached to the aortic valve, first from the parasternal long axis view (A-B), where the left atrium (LA), left ventricle (LV), and aortic root (Ao root) are noted. The mass (circle) is also seen from the parasternal short axis view (C-D), where the left atrium (LA), right atrium (RA), and right ventricle (RV) are also noted.

**Figure 2:**
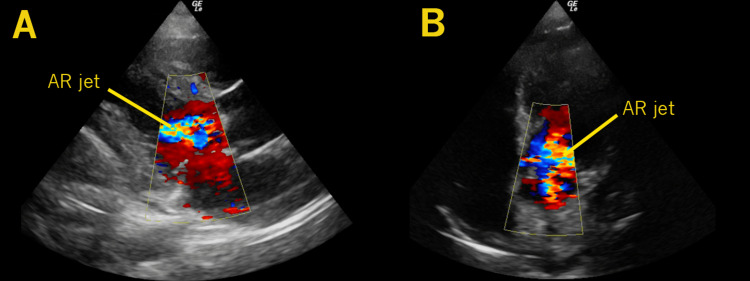
Aortic Regurgitation A parasternal long-axis view is seen with color Doppler over the aortic valve, demonstrating a turbulent aortic regurgitant jet (AR jet) (A). A similar finding is seen from the apical 5-chamber view (B).

A POCUS of the heart revealed a large, mobile mass on the aortic valve, highly suggestive of endocarditis (Figures 1-2, Video 1). Given these findings, blood cultures were obtained, broad-spectrum antibiotics were initiated, and general resuscitative and supportive measures were provided. As the diagnosis indicated a need for specialized cardiovascular and surgical care beyond the capabilities of the presenting facility, he was transferred to a tertiary center. Comprehensive echocardiography confirmed a 1.2 by 1.2 cm vegetation on his bicuspid aortic valve. His blood cultures were positive for methicillin-susceptible Staphylococcus aureus (MSSA). After a complicated hospital course, he unfortunately passed away.

**Video 1:** Case 1 Aortic Valve

Case 2

A 33-year-old male presented with one week of fatigue, fever, shortness of breath, and chest heaviness. His medical history included intravenous (IV) drug use and prior IE requiring bioprosthetic aortic and pulmonic valve replacements. On arrival, his vital signs were HR 138, BP 122/64, RR 20, T 38.9°C, and SpO₂ 94%. He appeared unwell, with a diastolic heart murmur and diminished breath sounds on auscultation. A petechial rash was noted on his lower extremities.

**Figure 3:**
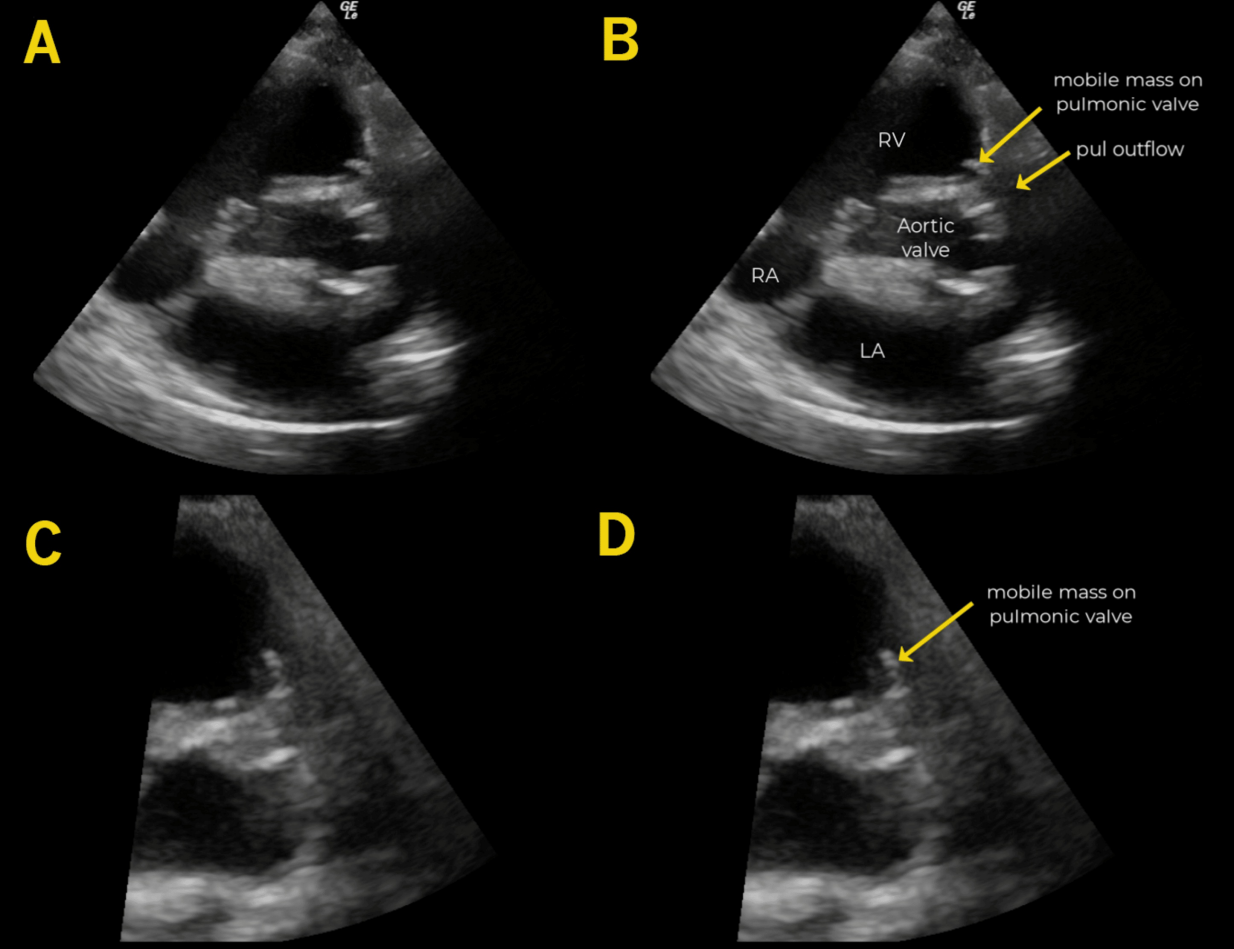
Pulmonic Valve Vegetation In these parasternal short-axis views taken at approximately the aortic valve level, an echogenic mobile mass (arrow) is seen attached to the pulmonic valve. It is even more prominent in the zoomed-in images (C-D). The right atrium (RA), right ventricle (RV), left atrium (LA), aortic valve, and pulmonic outflow are noted (B).

Given the clinical suspicion for IE, cardiac POCUS was performed, revealing a highly mobile mass on the pulmonic valve (Figure 3, Video 2). As this presentation occurred at an academic referral center, the patient was able to receive definitive management locally. Notably, the POCUS images obtained in the ED were archived in the hospital PACS (Picture Archiving and Communication System), allowing early review by subspecialists before a comprehensive transthoracic echocardiogram (TTE) and transesophageal echocardiogram (TEE) were performed.

**Video 2:** Case 2 Pulmonic Valve

Resuscitative and supportive measures were initiated, and the patient was admitted for further management. TEE confirmed a 2 cm pulmonic vegetation as well as an aortic paravalvular abscess. His blood cultures were positive for MSSA. He underwent repeat aortic and pulmonic valve replacements, and after a prolonged hospital course, he was successfully discharged home.

Case 3

A 25-year-old female presented to a community hospital with hallucinations, engaging in conversations with individuals who were not present. Further history from the family revealed intermittent fevers that resolved with acetaminophen. She had a known history of IV drug use.

**Figure 4:**
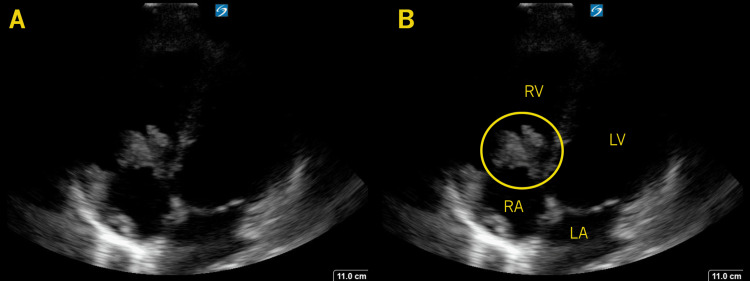
Tricuspid Valve Vegetation In this apical 4-chamber view, a large, echogenic mass (circle) is seen attached to the tricuspid valve. The right atrium (RA), right ventricle (RV), left atrium (LA), and left ventricle (LV) (B).

On examination, her vital signs were HR 152, BP 92/68, RR 16, T 37.8°C, and SpO₂ 90%. She appeared thin and unwell. No heart murmur was appreciated, and no skin changes were noted. Given her history of fevers and IV drug use, IE suspicion was raised. Cardiac POCUS revealed a large, irregular, mobile vegetation on the tricuspid valve (Figure 4, Video 3).

**Video 3:** Case 3 Tricuspid Valve

Based on these findings, general supportive and resuscitative measures were initiated, and the patient was immediately transferred to a center with comprehensive cardiac surgical, infectious disease, and behavioral health services. The TEE noted 3.8 by 1.2 cm vegetations on her tricuspid valve. Her blood cultures were positive for MSSA. She subsequently underwent tricuspid valve repair, completed a six-week course of IV antibiotics, and was ultimately discharged home.

Case 4

A 46-year-old male presented to a rural critical access hospital with several days of worsening right arm pain, swelling of the right hand, and discoloration of the 5th digit. He had a history of IV drug use.

On examination, his vital signs were within normal limits, and he was afebrile. Mild swelling of the right hand was noted, along with darkened discoloration of the 5th digit. The radial pulse was palpable, no heart murmurs were appreciated, and the skin was otherwise unremarkable.

**Figure 5:**
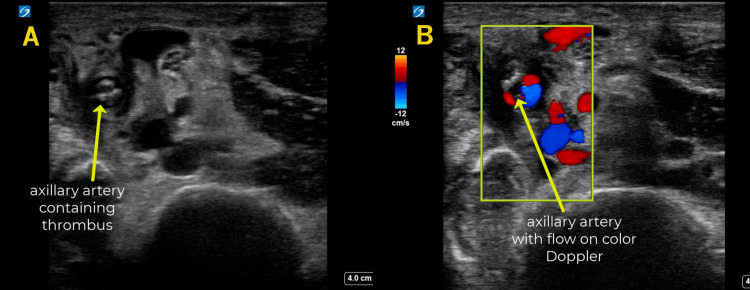
Partial Axillary Embolic Disease In these images taken from the proximal upper arm, the dilated axillary artery is seen containing irregular, echogenic thrombus (A); however, color Doppler evaluation confirms the occlusion is incomplete (B).

**Figure 6:**
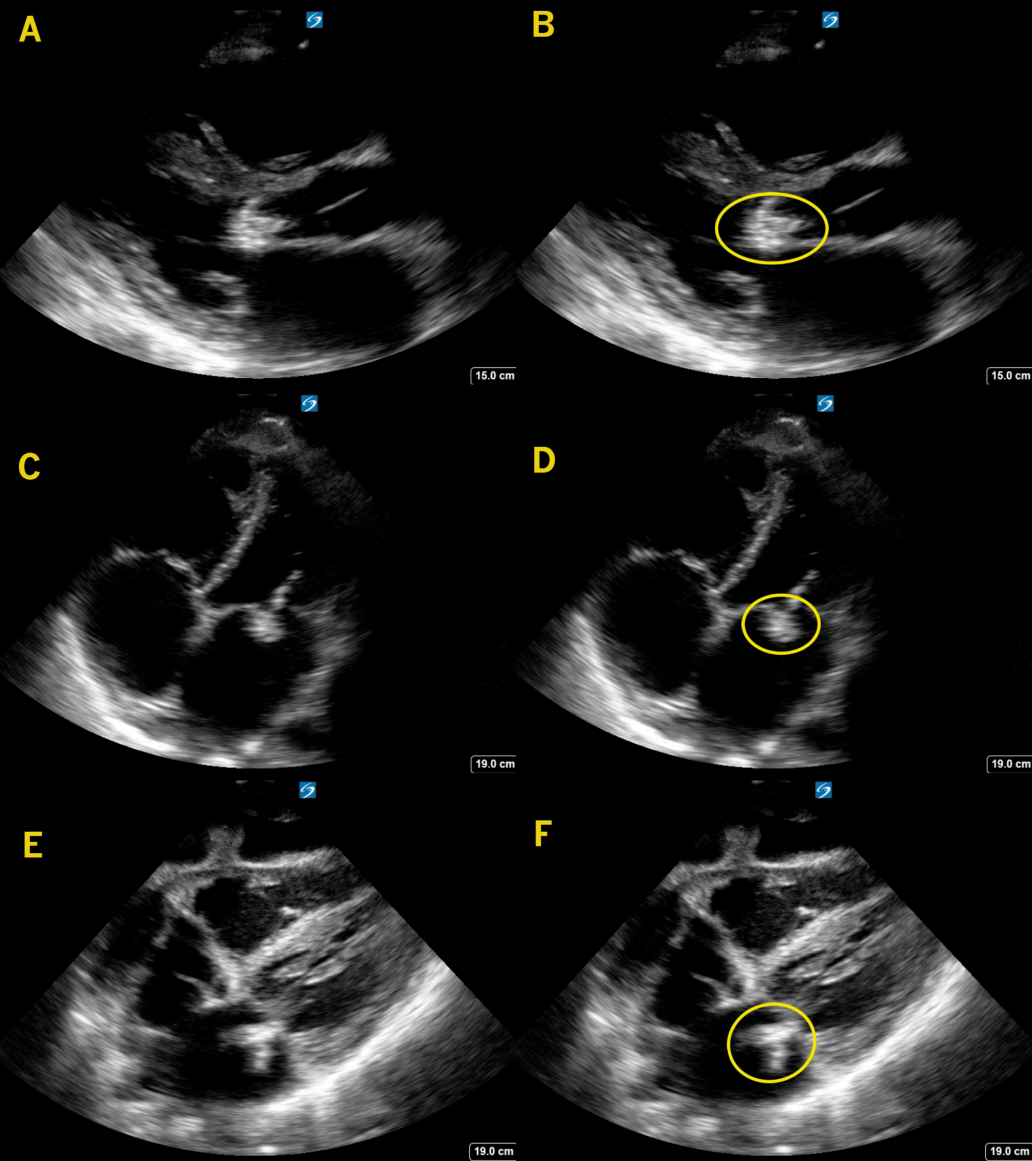
Mitral Valve Vegetation In these images, a large, echogenic mass (circle) is seen attached to the mitral valve from a parasternal long-axis view (A-B), an apical 4-chamber view (C-D), and a subcostal view (E-F). This mass is likely the origin of the embolic arterial disease seen in the axillary artery in image 5.

Given concerns for vascular compromise and possible cardiac origin, POCUS of both the heart and upper extremity was performed. Ultrasound revealed partial occlusion of the axillary and ulnar arteries, though pulsatile flow was still observed in the distal ulnar and radial arteries (Figure 5). Cardiac POCUS identified a large, irregularly shaped, highly mobile mass attached to the mitral valve, raising strong suspicion for IE (Figure 6, Video 4).

**Video 4:** Case 4 Mitral Valve

Blood cultures were obtained, broad-spectrum antibiotics were initiated, and arrangements were made for transfer to a higher-level facility for further management. However, the patient left the hospital against medical advice. Over the following months, he experienced a complicated clinical course but ultimately underwent mitral valve replacement and completed a prolonged course of IV antibiotics for endocarditis. His TEE prior to surgery noted a 1.8 by 0.8 cm vegetation on his mitral valve. His blood cultures were positive for *Streptococcus viridans*.

## Discussion

IE is a crucial problem, and timely diagnosis is essential to reduce complications and mortality [7]. Unfortunately, its initial symptoms - such as fever, fatigue, and malaise - are common in many ED presentations, making endocarditis a true diagnostic challenge. This reputation as the “great mimicker” underscores the need for heightened clinical suspicion in at-risk populations [8].

Given the nonspecific nature of early symptoms, emergency physicians must maintain a high index of suspicion, particularly in individuals with risk factors that increase their likelihood of developing IE. Early recognition is key, as prompt diagnosis allows for the initiation of targeted workup and involvement of appropriate specialists. By understanding the common signs and risk factors, emergency physicians can better identify patients who warrant a focused bedside ultrasound evaluation, rather than relying solely on later-stage clinical deterioration to raise concern. Each case brings some unique insight into the varied clinical manifestations and course of IE. All four of these cases have in common that the patients all had a history of IV drug use. IE cases continue to rise in the population, likely attributed to the increased use of IV drugs [4].

The first case presents the two most common symptoms of endocarditis, which are fever and murmur. POCUS allowed a definitive diagnosis to be made in minutes, expediting the decision for transfer to a higher level of care. As most rural or smaller community emergency physicians are aware, demonstrable and objective evidence of a definitive diagnosis and need for subspecialty services is useful in prioritizing and mobilizing resources for critical patient transfers, whereas more speculative or provisional diagnoses may be deprioritized pending further diagnostic evaluation. This case brought in two risk factors that are very common in left-sided IE: history of bicuspid valve and IV drug use. Three-fourths of patients with IE will have a pre-existing structural cardiac abnormality, examples being valvular disease from rheumatic heart disease, mitral valve prolapse with regurgitation, aortic stenosis, or congenital heart disease [9]. This case also noted a large vegetation which has been shown to have higher complication and mortality rates, particularly when they are over 10 mm in size [10], which unfortunately ultimately happened in this case.

The second case also presented with typical signs and symptoms of endocarditis and had some strong risk factors, including prior history of endocarditis, prosthetic valve repair, and history of IV drug use. This case again reiterated the point of early diagnosis to expedite transfer to tertiary care. With the diagnosis essentially confirmed, the subspecialists, such as cardiothoracic surgery, were involved early in the course rather than awaiting TEE, which may require 48 hours or more, depending on the facility and day of the week. With right-sided IE, the tricuspid valve is most frequently involved with isolated pulmonary valve (such as in the third case), involvement being <2% [11]. With right-sided IE, IV drug use, intracardiac devices, and central venous catheters are significant risk factors [12].

The third case had the least clear initial clinical presentation, with the lack of skin changes or an audible murmur. The history of IV drug use was the primary risk factor that might prompt consideration of endocarditis. The early incorporation of POCUS, with the findings as noted, provided the clinical conviction necessary to initiate aggressive evaluation, therapy, and management decisions, specifically three sets of blood cultures, initiation of broad-spectrum antibiotics, and early involvement of subspecialty services. Like the second case, right-sided IE has been highly associated with IV drug use, as in this case. With no other symptoms, one study found that 13% of intravenous drug abusers with fever were found later to have IE [11]. A “tricuspid syndrome” has been noted with persistent fever with pulmonary events, anemia, and microscopic hematuria in tricuspid IE [10].

The fourth and final case brings up a common scenario when an individual with a history of IV drug use presents with limb pain and/or swelling. Though this patient had signs that might suggest septic emboli, less critical diagnoses such as limb infections and thrombophlebitis are common in these individuals. Without overt red flags and without ready access to POCUS at the bedside, it would be conceivable to attribute these symptoms to less critical disease, delay definitive and accurate diagnosis, increasing the risk of complications and even death, particularly in patients with large vegetations such as this one.

Physicians should consider performing POCUS to evaluate for endocarditis in patients with fever and known or suspected risk factors, of which IV drug use is primary, but other risk factors may include implanted medical devices, known valvular abnormalities, or recent medical instrumentation. Additionally, patients with findings that are highly suggestive of endocarditis, including characteristic skin changes or embolic phenomena, should be given consideration for cardiac POCUS. POCUS not only provides information to confirm suspected endocarditis but also allows evaluation of valvular damage and dysfunction. The authors have experienced cases where, based on POCUS findings, upon review by and in consultation with subspecialists, provisional plans for non-operative care can be made, allowing patients to be managed locally even in a center where complex valvular interventions are not performed. Though this involves more specialized skills and is a unique scenario, it nonetheless demonstrates the high-level decision-making that may be provided by clinicians armed with mastery of the powerful bedside tool of POCUS.

To evaluate for IE on POCUS and improve the likelihood of visualizing vegetations, if present, optimal windows and views must be sought. If possible, imaging from the left lateral decubitus position can improve cardiac POCUS views. Optimizing the patient's respiratory phase can further enhance views. Additionally, the zoom function further improves views by increasing spatial, contrast, and temporal resolution. Systematic and intentional fanning or sweeping movements to more thoroughly evaluate each valve will increase the likelihood of identifying vegetations. Evaluating valves from every possible view and in short and long axes. Lastly, color Doppler interrogation may reveal regurgitation, which may be a secondary sign of endocarditis, though not definitive [13]. More experienced POCUS users may also estimate regurgitant severity, leading to potential decisions regarding surgical needs and disposition.

The vegetations which are characteristic of IE appear as highly mobile echogenic masses, typically gray scale color similar to myocardium, originating on the upstream side of valvular leaflets flanking the path of the jet. They typically prolapse into the upstream chamber and have orbiting/chaotic movement. Other findings, such as valvular abscesses, may be seen as well and are found on the adjacent walls of the myocardium near the valve. Recognition of vegetations requires some experience and requisite knowledge of the normal appearance of the valves throughout the cardiac cycle, including some of the less pathologic variations, such as mitral annular calcification and stenotic aortic calcifications.

## Conclusions

IE remains a challenging diagnosis in the ED, often presenting with nonspecific common symptoms that can lead to delays in treatment. The cases presented in this series highlight the variability in clinical presentation, the importance of maintaining a high index of suspicion, and the critical role of bedside ultrasound in early diagnosis. By utilizing POCUS, emergency physicians can identify endocarditis more rapidly, expedite appropriate management decisions, and facilitate timely consultation with specialists. In rural and resource-limited settings, early POCUS findings may also help streamline transfer decisions, ensuring that critically ill patients receive definitive care as soon as possible. While comprehensive echocardiography remains the gold standard for diagnosis, POCUS serves as a valuable adjunct, particularly in patients with high-risk features such as intravenous drug use, unexplained fever, or embolic phenomena. Further research is needed to establish standardized POCUS protocols for endocarditis evaluation, but the integration of bedside ultrasound into emergency practice has the potential to improve diagnostic accuracy, optimize resource utilization, and ultimately enhance patient outcomes.
